# Supplementary material for: Reconstructing the quantum critical fan of strongly correlated systems using quantum correlations
Source: Nat Commun. 2019 Feb 4;10:577. doi: 10.1038/s41467-019-08324-9 (PMC6362001; doi:10.1038/s41467-019-08324-9)
Supplement: Supplementary file 1 — Supplementary Information [file 41467_2019_8324_MOESM1_ESM.pdf]

Supplementary Information
Reconstructing the quantum critical fan of strongly correlated systems using quantum correlations

Irénée Frérot*[1,2], and Tommaso Roscilde†[2,3]
[1] *ICFO-Institut de Ciencies Fotoniques, The Barcelona Institute of Science and Technology, Av. Carl Friedrich Gauss 3, 08860 Castelldefels (Barcelona), Spain.*
[2] *Univ Lyon, Ens de Lyon, Univ Claude Bernard, CNRS, Laboratoire de Physique, F-69342 Lyon, France. and*
[3] *Institut Universitaire de France, 103 boulevard Saint-Michel, 75005 Paris, France*
(Dated: November 23, 2018)

SUPPLEMENTARY NOTE 1: QUANTUM COHERENCE LENGTH VS. CORRELATION LENGTH AROUND THE QCP

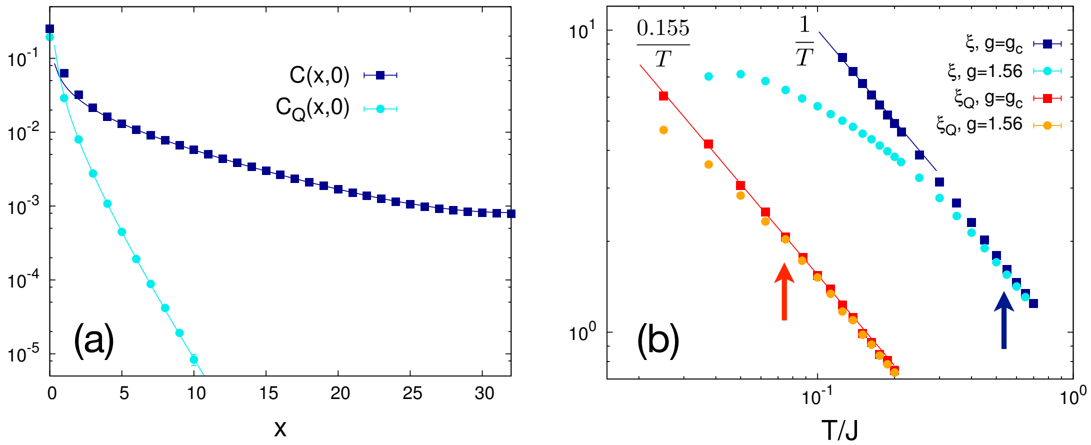

SUPPLEMENTARY FIG. 1: **Ordinary vs. quantum correlations for the 2d transverse-field Ising model.** (a) Ordinary correlation function (dark-blue squares) vs. quantum correlation function (light-blue circles). The model parameters are $g = g_c$, $T/J = 0.1$ and $L = 64$. The solid lines show fits to the form Eq. (2); (b) Temperature scaling of the correlation length (ξ, blue) and quantum coherence length (ξ_Q, red and yellow) along the quantum critical trajectory ($g = g_c = 1.522...$, squares) of the 2d transverse-field Ising model, and slightly above ($g = 1.56$, circles). The solid lines show the low-T behavior as $\sim 1/T^{1/z}$ along the quantum critical trajectory – notice the wide separation between ξ and ξ_Q. The arrows show the temperatures around which ξ (blue arrow) and ξ_Q (red arrow) at $g = 1.56$ depart from the same curves for $g = g_c$.

In this section we discuss the scaling of the correlation length and of the quantum coherence length in the QC regime of the $2d$ transverse field Ising model. We extract the correlation length ξ and the quantum coherence length ξ_Q by fitting the ordinary correlation and the quantum correlation function [1]:

$$\begin{aligned} C(\mathbf{r}) &= \langle S_i^z S_{i+\mathbf{r}}^z \rangle \\ C_Q(\mathbf{r}) &= \langle S_i^z S_{i+\mathbf{r}}^z \rangle - \frac{1}{\beta}\int_0^\beta d\tau \langle S_i^z(\tau) S_{i+r}^z(0)\rangle \ . \end{aligned} \quad (1)$$

where $S_i^z(\tau) = e^{\tau\mathcal{H}} S_i^z e^{-\tau\mathcal{H}}$ is the imaginary-time evolved operator. For separations $\mathbf{r} = (x, 0)$ both functions can be fitted to the form

$$f(x) = a\left(\frac{e^{-x/\xi_{(Q)}}}{x^b} + \frac{e^{-(L-x)/\xi_{(Q)}}}{(L-x)^b}\right) \ . \quad (2)$$

An example of the fits are shown in Supplementary Figure 1(a), highlighting as well the vast difference in range between ordinary and quantum correlations. The results of the fit of the above correlation functions along the quantum critical trajectory

* irenee.frerot@icfo.eu
† tommaso.roscilde@ens-lyon.fr

($g = g_c$) are shown in Supplementary Figure 1(b). There it is shown that both ξ and ξ_Q scale as $T^{-1/z}$ (with $z = 1$) at low temperatures – namely within the QC regime. In addition to the results for the quantum critical trajectory, we show the results for $g = 1.56 > g_c$, namely for a transverse field slightly above the quantum critical value. We observe that the T-dependence of the correlation length ξ is extremely sensitive to the departure from the critical point, separating from the $g = g_c$ curve at a temperature ($T^* \approx 0.4J$) higher than the gap (estimated to be $\Delta \approx 0.1J$), to saturate to a finite value $\approx c/\Delta$ when $T \lesssim \Delta$. As a consequence, despite the small deviation from g_c, ξ loses all traces of the scaling as T^{-1} characteristic of the QC regime. On the other hand the quantum coherence length for $g = 1.56$ follows the same scaling as that at $g = g_c$ down to much lower temperatures ($T^*_Q \approx 0.06J$), and this opens a finite temperature window over which the T^{-1} scaling is manifest – the same observation holds for the quantum variance, exhibiting the $T^{-\psi}$ scaling over the same temperature range (see main text). As already mentioned in the main text, this observation can be traced back to the fact that in the QC regime $\xi_Q \approx c_Q/T \ll \xi \approx c/T$, and hence, for $g > g_c$, the finiteness of $\xi \approx c/\Delta$ at $T = 0$ leads to a deviation from the QC scaling at a temperature $T^* \approx \Delta$; but, as $\xi_Q = \xi$ at $T = 0$, the QC scaling of ξ_Q persists down to $T^*_Q \approx (c_Q/c)\, T^* \ll T^*$. This justifies the ability of quantum fluctuations to detect a QC regime of finite extent in temperature even when the ground state is quantum disordered. In the regime exhibiting an ordered ground state, the explanation of the sensitivity of quantum fluctuations to the QC regime is instead to be attributed to the weakness of the singularity exhibited by the QV at any thermal transition. As a consequence the Ginzburg region for quantum fluctuations is extremely small, and it affects minimally the temperature scaling of the QC regime above T_c.

SUPPLEMENTARY NOTE 2: TEMPERATURE SCALING OF THE QUANTUM VARIANCE FOR THE HEISENBERG BILAYER

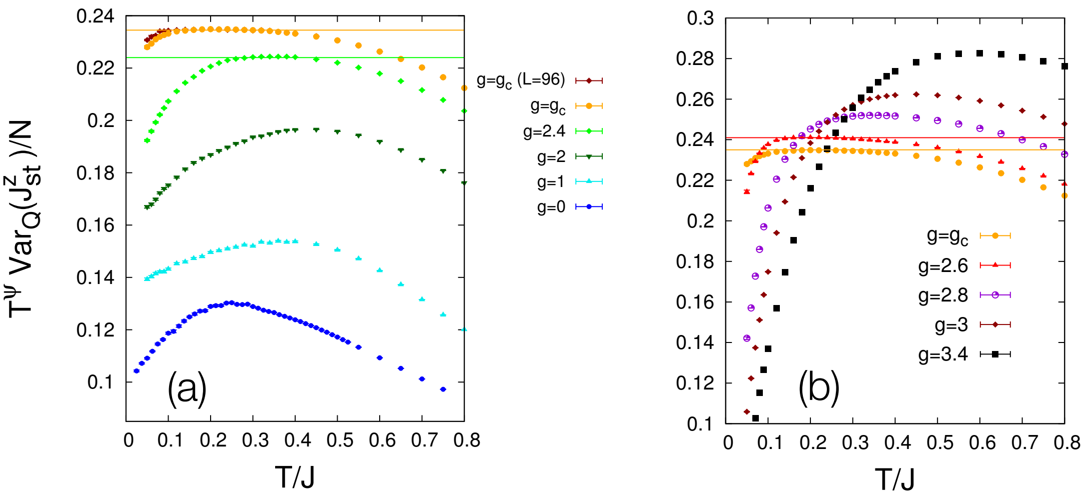

SUPPLEMENTARY FIG. 2: **Temperature scaling of the order-parameter quantum variance in the S=1/2 Heisenberg bilayer.** Both panels show the temper-ature dependence of $T^{\psi}\mathrm{Var_Q}(J^z_{\rm st})/N$ for a $N = L \times L \times 2$ lattice, below (a) and above (b) the quantum critical point. All data are for a $L = 64$ lattice, except for the data in (a) at $g = g_c$ and $L = 96$. The horizontal solid lines mark the plateaus exhibited by the curves in the quantum-critical regime. The low-T downturn of the data for $g = g_c$ is a finite-size effect, as shown by the comparison between the data for $L = 64$ and $L = 96$ in (a).

In this section we discuss the temperature scaling of the order-parameter quantum variance for the Heisenberg bilayer and the specific signatures of the QC regime. Supplementary Figure 2 shows the function $T^{\psi}\mathrm{Var_Q}(J^z_{\rm st})/N$ for various values of the g parameter below and above the QCP. In the vicinity of the QCP this function shows an extended plateau, manifesting the existence of a clear temperature interval characterized by the thermal QC scaling $\sim T^{\psi}$ – the QC regime is then identified by this feature and the additional constraint that the prefactor to the $T^{-\psi}$ scaling must be close (within an ϵ tolerance) to that exhibited along the QC trajectory.

The plateau transforms into a broad maximum when leaving the QC regime – namely the quantum variance exhibits a $T^{-\psi}$ temperature scaling only locally in T (as its logarithmic derivative varies continuously with T). This is evident also in the single-layer limit $g = 0$, reproducing the physics of the $S = 1/2$ $2d$ Heisenberg antiferromagnet. We conclude that the latter model does *not* show any evidence of an extended region of $T^{-\psi}$ scaling for the order-parameter quantum variance: namely quantum fluctuations do not detect any sign of a quantum-critical/renormalized-classical crossover in this system.

SUPPLEMENTARY NOTE 3: QUANTUM VARIANCE VS. QUANTUM FISHER INFORMATION IN THE INFINITE-DIMENSIONAL QUANTUM ISING MODEL

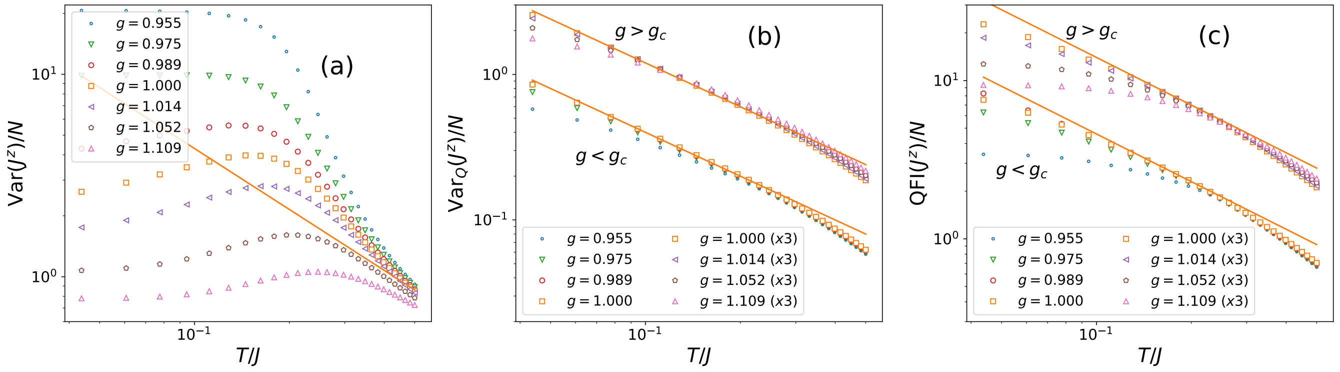

SUPPLEMENTARY FIG. 3: **Infinite-range transverse-field Ising model.** Temperature scaling of the variance (a), quantum variance (b) and quantum Fisher information (c) of the transverse field Ising model in $d = \infty$. The data are obtained for a system size of $N = 1000$.

In this section we show the temperature scaling of the order parameter fluctuations, quantum variance and quantum Fisher information of the order parameter for the quantum Ising model $d = \infty$, namely the fully connected Ising model, whose Hamiltonian reads

$$\mathcal{H}/\mathcal{J} = -\frac{(J^z)^2}{N} - gJ^x \tag{3}$$

Here we indicate with $\mathcal{J}$ the spin-spin coupling energy, not to be confused with the length of the collective spin $\mathbf{J} = \sum_i \mathbf{S}_i$. This model can be exactly solved by diagonalizing the Hamiltonian in each of the sectors defined by the conservation of $\mathbf{J}^2 = \hbar J(J+1)$, namely by diagonalizing the reduced Hamiltonian matrices $\mathcal{H}^{(J)}_{M,M'} = \langle JM|\mathcal{H}|JM'\rangle$. This analysis gives readily access to the eigenvalues E_n and, when supplemented with the formula for the degeneracy of each J sector [2], to all observables related to the collective spin. From this one can readily reconstruct the order parameter variance $\mathrm{Var}(J^z)$, the quantum variance of the order parameter [3]

$$\mathrm{Var}_\mathrm{Q}(J^z) = \sum_{nm} \left(\frac{p_n + p_m}{2} - \frac{p_n - p_m}{\log(p_n/p_m)} \right) |\langle n|J^z|m\rangle|^2 \tag{4}$$

and its quantum Fisher information [4]

$$\mathrm{QFI}(J^z) = \sum_{nm} 2\frac{(p_n - p_m)^2}{p_n + p_m} |\langle n|J^z|m\rangle|^2 \tag{5}$$

where $p_n = \exp(-\beta E_n)/\mathcal{Z}$ with $\mathcal{Z}$ the partition function, and we have assumed that $\langle J^z \rangle = 0$ (valid on a finite-size system). Introducing the two functions

$$\begin{aligned} G_\mathrm{QV}(x,y) &= \frac{x+y}{2} - \frac{x-y}{\log(x/y)} \\ G_\mathrm{QFI}(x,y) &= 2\frac{(x-y)^2}{x+y} \end{aligned} \tag{6}$$

it is easy to show that $G_\mathrm{QV}(x,y) \leq G_\mathrm{QFI}(x,y)/4 \leq 3G_\mathrm{QV}(x,y)$ over the square $\{x \in [0,1], y \in [0,1]\}$. This inequality chain is then inherited by the quantum variance and quantum Fisher information, namely

$$\mathrm{Var}_\mathrm{Q}(J^z) \leq \mathrm{QFI}(J^z)/4 \leq 3\mathrm{Var}_\mathrm{Q}(J^z) \ . \tag{7}$$

This implies *e.g.* that the quantum critical divergence of the quantum Fisher information at $g = g_\mathrm{c}$ (for $T \to 0$ and for $N \to \infty$) is identical to that of the quantum variance. Supplementary Figure 3 shows the temperature scaling of these three quantities

around the quantum critical point of the model at $g = g_c = 1$ for a system of size $N = 1000$. Similarly to what seen in the main text for the case $d = 2$, we observe that the T-dependence of the total variance strongly depends on g around the quantum critical point. Moreover finite-size effects in this infinite-connectivity model are so strong that the expected power-law scaling along the quantum critical trajectory as $T^{-\psi}$ with $\psi = 1$ (for mean-field exponents) is not observed. On the other hand, finite-size effects are much weaker on the quantum variance, which clearly exhibits the above power-law scaling at $g = g_c$; and, similarly to $d = 2$, the same scaling is observed as well in an intermediate temperature range for $g \gtrsim g_c$ and $g \lesssim g_c$, marking the signature of the quantum critical fan. A rather similar behavior is also seen in the T-dependence of the quantum Fisher information, although the latter appears to be more affected by finite-size effects; and it seems to be more sensitive to the deviation from the quantum critical point, suggesting a smaller quantum critical fan. We notice that our calculation of the quantum Fisher information differs from that of Ref. [5] in that the latter was limited to the Hamiltonian sector $J = N/2$, making the model identical to that of the bosonic Josephson junction [6].

SUPPLEMENTARY NOTE 4: QUANTUM VARIANCE VS. QUANTUM FISHER INFORMATION IN THE ONE-DIMENSIONAL QUANTUM ISING MODEL

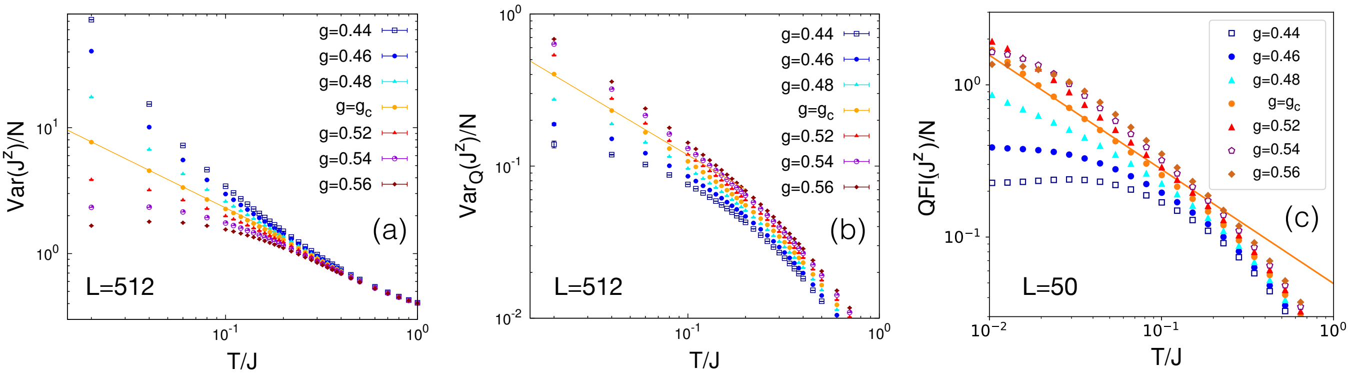

SUPPLEMENTARY FIG. 4: **Transverse-field Ising chain.** Temperature scaling of the variance (a), quantum variance (b) and quantum Fisher information (c) of the transverse field Ising model in $d = 1$. The data in (a) and (b) were obtained via quantum Monte Carlo for a chain of length $L = 512$ with periodic boundary conditions. The data in (c) were calculated for a chain of length $L = 50$ with open boundary conditions.

We conclude by discussing the case $d = 1$, which can be solved exactly via a Jordan-Wigner transformation to free fermions, and which exhibits a quantum critical point for $g = g_c = 1/2$. The $1d$ case is special in that the system no-longer exhibits a finite-T transition on the ordered side $g < g_c$. The exact solution gives access to the dynamical susceptibility χ'' for the order parameter, which allows then to reconstruct the quantum Fisher information via the formula of Ref. [5]. At finite temperature the exact solution is only viable for open boundary conditions, and it remains rather laborious (implying the calculation of Pfaffians of large matrices) in order to reconstruct correlation functions. It is therefore useful to make use of the numerical solution via quantum Monte Carlo for the quantities which are accessible to this method, namely the total and quantum variance.

The temperature dependence of the latter two quantities is shown in Supplementary Figure 4(a-b) for a chain of length $L = 512$ with periodic boundary conditions. There we observe that a quantum-critical $T^{-\psi}$ scaling (with $\psi = 3/4$) is clearly exhibited by the variance along the quantum critical trajectory for $T/J \lesssim 0.1$. Yet the total variance shows also a strong sensitivity to a deviation from the quantum critical point, and, over the above temperature range, the power-law behavior is quickly lost when g deviates from g_c. The quantum variance shows less sensitivity to a deviation from g_c, but, contrary to $d > 1$, its power-law scaling along the quantum critical trajectory shows up on a smaller temperature range than for the total variance; as a consequence, in the data presented in Supplementary Figure 4 the quantum critical behavior of the quantum variance is not observed away from g_c. We also remark that the $d = 1$ case is rather special for the quantum Ising model in that, over the temperature range shown in the figure, the quantum variance is not maximal along the quantum critical trajectory (compare Supplementary Figure 3(b), and Figure 2(b) of the main text), but rather for $g > g_c$.

Interestingly all the remarks made for the quantum variance also hold for the quantum Fisher information, shown in Supplementary Figure 4(c) for a open-boundary chain of length $L = 50$. We remark here that, because of the difference in system size and boundary conditions, the inequality Eq. (7) between the quantum Fisher information and the quantum variance does not hold at all temperatures between the data in Supplementary Figure 4(b) and (c) (but it does for data of equal size and same boundary conditions).

We conclude therefore that, in the specific case of the 1d quantum Ising model, the temperature scaling of quantum correlations does not exhibit a quantum critical regime which is significantly broader in g-T space than that shown by conventional

correlations, and which for both forms of correlations appears to be tightly confined around the quantum critical trajectory. The upper bound to the $T^{-\psi}$ quantum critical scaling of the total variance along such a trajectory ($T/J \approx 0.1$) is consistent with the upper bound to the QC regime exhibited by the temperature scaling of the free energy [7].

---

[1] D. Malpetti and T. Roscilde, "Quantum correlations, separability, and quantum coherence length in equilibrium many-body systems," *Phys. Rev. Lett.* **117**, 130401 (2016).

[2] F. T. Arecchi, E. Courtens, R. Gilmore, and H. Thomas, "Atomic coherent states in quantum optics," *Phys. Rev. A* **6**, 2211–2237 (1972).

[3] I. Frérot and T. Roscilde, "Quantum variance: A measure of quantum coherence and quantum correlations for many-body systems," *Phys. Rev. B* **94**, 075121 (2016).

[4] L. Pezzé and A. Smerzi, "Quantum theory of phase estimation," in *Atom Interferometry, Proceedings of the International School of Physics "Enrico Fermi", Course 188* (G. M. Tino and M. A. Kasevich, eds.), Amsterdam: IOS Press, 2014.

[5] P. Hauke, M. Heyl, L. Tagliacozzo, and P. Zoller, "Measuring multipartite entanglement via dynamic susceptibilities," *Nature Physics* **12**, 778–782 (2016).

[6] L. Pezzè, A. Smerzi, M. K. Oberthaler, R. Schmied, and P. Treutlein, "Quantum metrology with nonclassical states of atomic ensembles," *Rev. Mod. Phys.* **90**, 035005 (2018).

[7] A. Kopp and S. Chakravarty, "Criticality in correlated quantum matter," *Nature Physics* **1**, 53–56 (2005).
